# Early prediction of new-onset physical disability after intensive care unit stay: a preliminary instrument

**DOI:** 10.1186/s13054-014-0455-7

**Published:** 2014-07-31

**Authors:** Anna Schandl, Matteo Bottai, Ulrika Holdar, Elisabeth Hellgren, Peter Sackey

**Affiliations:** Department of Anesthesiology, Surgical Services and Intensive Care Medicine, Karolinska University Hospital Solna, 17176 Stockholm, Sweden; The Institution of Physiology and Pharmacology, Section for Anesthesiology and Intensive Care Medicine, Karolinska Institutet, 17177 Stockholm, Sweden; The Unit of Biostatistics, Institute of Environmental Medicine, Karolinska Institutet, 17177 Stockholm, Sweden

## Abstract

**Introduction:**

Many intensive care unit (ICU) survivors suffer from physical disability for months after ICU stay. There is no structured method to identify patients at risk for such problems. The purpose of the study was to develop a method for early in-ICU prediction of the patient’s individual risk for new-onset physical disability two months after ICU stay.

**Methods:**

In total, 23 potential predictors for physical disability were assessed before individual ICU discharge. Two months after ICU discharge, out of 232 eligible patients, 148 ICU survivors (64%) completed the activity of daily living (ADL) staircase questionnaire to determine new-onset physical disability.

**Results:**

A total of 95% percent of patients had no ADL reduction prior to ICU admission. Forty-seven percent (*n* = 69) of questionnaire responders suffered from worsened ADL. We identified four independent predictors for new-onset physical disability: Low educational level (odds ratio (OR) = 6.8), impaired core stability (OR = 4.6), fractures (OR = 4.5) and ICU length of stay longer than two days (OR = 2.6). The predictors were included in a screening instrument. The regression coefficient of each predictor was transformed into a risk score. The sum of risk scores was related to a predicted probability for physical disability in the individual patient. The cross-validated area under receiver operating characteristics curve (AUC) for the screening instrument was 0.80.

**Conclusions:**

Educational level is the single most important predictor for new-onset physical disability two months after ICU stay, followed by impaired core stability at ICU discharge, the presence of fractures and ICU stay longer than two days. A simple screening instrument based on these predictors can be used at ICU discharge to determine the risk for new-onset physical disability. This preliminary instrument may help clinicians to identify patients in need of support, but needs external validation prior to wider clinical use.

**Electronic supplementary material:**

The online version of this article (doi:10.1186/s13054-014-0455-7) contains supplementary material, which is available to authorized users.

## Introduction

More than five million people are treated for life-threatening illness or injury in intensive care units (ICUs) in the United States annually [1]. A significant proportion of ICU survivors report long-term physical and psychological problems that may lead to impaired daily functioning [2], delayed return to work [3] and reduced quality of life [4]. To improve long-term outcomes, different ICU follow-up programs have been suggested for patients with long ICU length of stay [5] or for those deemed in need [3]. So far, the appropriateness of such patient selection, as well as the efficacy of interventions after ICU discharge to improve outcome, such as ICU follow-up clinics and home-based rehabilitation programs, is uncertain [6,7]. Including patients at high risk for new-onset morbidity after ICU stay, rather than all available patients, would increase the chance of revealing treatment effects in interventional studies [8,9]. Additionally, follow-up of high-risk patients would likely be more cost-beneficial than follow-up for all patients. The trajectory of recovery may potentially be improved by early identification and rehabilitation in risk patients. The aim of this study was to develop a method for early in-ICU prediction of the patient’s individual risk for new-onset physical disability two months post-ICU.

## Materials and methods

### Study design

We conducted a prospective cohort study to assess, at ICU discharge, the relative contribution of potential predictors for later physical disability. The patients were recruited from a 13-bed general ICU, at a tertiary care hospital serving as a trauma referral center for patients from the metropolitan Stockholm area. Around 900 adult medical, surgical and trauma patients are admitted to the general ICU yearly. The study was approved by the Regional Ethical Review Board in Stockholm (2010/206-31/1) and written informed consent was obtained from all participants.

### Participants

**Figure 1:**
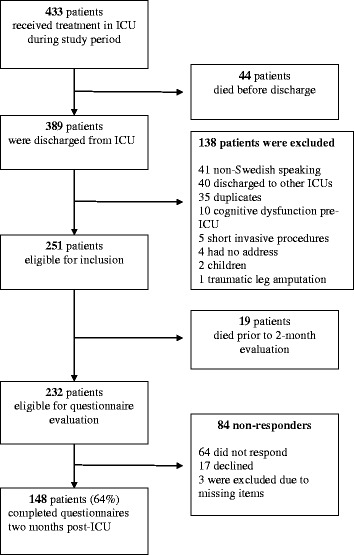
**Flow chart of patient inclusion.**

During a six-month period in 2011 all patients, independent of ICU length of stay, were consecutively enrolled in the study at ICU discharge. Eligible patients were those who consented to participate in the study and were discharged to a ward (that is not transferred to other ICUs during their illness) (Figure 1). Patients were excluded if they were admitted shortly solely for invasive procedures (such as placement of epidural catheters or central venous lines) or if they were considered unable to fill in the outcome questionnaires due to pre-existing cognitive dysfunction. Non-Swedish-speaking patients as well as patients with no formal address were also excluded. For patients readmitted to the ICU, only data from the final admission was used. The participants in this study were included in parallel in a study predicting psychological problems [10].

### Data collection

#### Selection of potential predictors

The potential predictors were selected through a review of previous studies investigating risk factors for physical morbidity after critical illness, together with studies describing risk factors influencing physical recovery in general. An additional file shows the identified risk factors in the literature review in more detail (see Additional file 1). The result of the review was presented to ICU clinicians running a follow-up clinic: doctors, nurses, physiotherapists, an occupational therapist and a clinical psychologist. Risk factors from the review were discussed, as well as the feasibility of measuring them in everyday clinical practice. Potential predictors considered reasonably reproducible in most patients and fairly easy to assess at discharge by ICU clinicians in a heterogeneous critically ill population were included [11]. After reaching consensus 23 potential predictors, or feasible proxy measures, of later physical disability were selected for evaluation at ICU discharge (Table 1).

**Table 1:** **Description of potential predictors, patient and treatment characteristics for patients reporting new-onset physical disability or no new-onset physical disability two months after ICU discharge and the predictors’ univariate associations**

**Predictors**	**Categorization**	**Physical disability (n = 69)**	**No physical disability (n = 79)**	**Univariate associations (** ***P*** **values)**
Age		59 ± 17	51 ± 17	<0.01*
Gender	Men	59%	64%	>0.1
	Women	41%	36%	
Marital status	Single	37%	39%	>0.1
	Life partner	63%	61%	
Education level	Elementary school	32%	8%	<0.001*
	Senior high school/College/University	68%	92%	
Occupational status pre-ICU	Sick leave or unemployed	12%	21%	>0.1
ICU length of stay >2 days		57%	29%	<0.001*
SAPS 3		59 ± 20	51 ± 16	<0.1*
Diagnosis	Trauma	26%	22%	>0.1
	Surgery	28%	32%	
	Medical diseases	13%	23%	
	Infection	32%	22%	
Somatic comorbidity	0	35%	57%	<0.1*
(Charlson Comorbidity Index^1^)	1	24%	16%	
	≥2	41%	27%	
Psychological problems pre-ICU		22%	19%	>0.1
Propofol administered for		16%	16%	>0.1
>24 hours				
Midazolam administered for		24%	10%	<0.05*
>24 hours				
Morphine use (days)		1.3 ± 3.3	1.0 ± 2.8	>0.1
Ventilator treatment for >24 hours		38%	18%	>0.1
Delirium		32%	18%	<0.05*
Fractures		22%	4%	<0.001*
(arm, leg, pelvis, costae, dorsal)				
Body mass index (BMI)	Underweight	4%	5%	>0.1
	Normal weight	25%	30%	
	Overweight	43%	49%	
	Obesity	26%	14%	
Supplemental oxygen		29%	19%	>0.1
>3 liters/minute				
Grip strength		13%	6%	>0.1
(Not capable of holding a glass)				
Core instability		60%	19%	<0.001*
(Not capable of sitting independently)				
Ability to initiative		32%	16%	<0.01*
(takes no own initiative in ICU)				
Depressive symptoms		34%	26%	>0.1
(appears depressed in ICU)				
Lack of social support		13%	11%	>0.1
(no family present in ICU)				

*Included in the multivariate logistic regression model. ^1^Charlson Comorbidity Index was originally developed to predict the 10-year mortality for patients with somatic diseases such as heart disease or cancer. Each condition is assigned a score of 1, 2, 3 or 6, depending on the mortality risk associated with this condition. Underweight = BMI <18.5, Normal weight = BMI 18.5 to 24.9, Overweight = BMI 25 to 29.5, Obesity = BMI >30. ICU, intensive care unit; SAPS 3, Simplified Acute Physiology Score 3.

#### Assessment of predictors

At ICU discharge, data were registered for each patient in a document, including the 23 potential predictors. Patient characteristics were noted; age, gender, marital status, educational level, occupational status, presence of pre-existing diseases and previous psychological problems were obtained from the medical charts and from the patient him/herself. Level of education was classified as low, intermediate and high education. Low educational level implied elementary school education only; intermediate educational level implied secondary, nonacademic education; and high educational level implied academic education. The Charlson Comorbidity Index (CCI) was used to estimate the burden of pre-existing somatic disease [12]. Patients were considered having previous psychological problems if they reported prior episodes of depression or anxiety, a psychiatric diagnosis was stated in the medical charts or if they had documented alcohol or drug abuse.

Data regarding patients’ ICU stay, Simplified Acute Physiology Score (SAPS) 3 calculated at ICU admission, main ICU diagnosis (divided into trauma, surgical diagnoses, medical diseases or infections), ICU length of stay, type and duration of sedative, opiate infusions, duration of invasive ventilator support, fractures, presence of delirium assessed with the Confusion Assessment Method for the Intensive Care Unit [13] and body mass index (BMI), were obtained from the local patient data management system and from the medical charts.

The patient’s nurse assessed and documented six potential risk factors at ICU discharge: Need for supplemental oxygen >3 liters/minute, reduced grip strength, poor core stability, inability to take own initiative, depressive symptoms and reduced social support during the ICU stay. The patient’s need for supplemental oxygen >3 liters/minute was noted when the patient was transferred from the ICU. Grip strength was assessed as the ability to hold a glass in one hand. The assessment was considered to have reasonable potential of being predictive of some activity of daily living (ADL) functions, such as eating or drinking. If the patient was unable to sit independently (without support of others) on the bedside of their ICU bed, he/she was considered having poor core stability. The patient was considered unable to take own initiative if he or she did not verbally or nonverbally take initiative to any activity, such as expressing the need to change position in bed, to drink water, eat food or make spontaneous requests for activities. Depressive symptoms (sadness, apathy or expressing feelings of hopelessness) were also noted. For communicative patients, the patient was asked if he/she felt depressed: ‘Do you feel down?’. Patients with no visits from family or next-of-kin during the ICU stay were classified as having reduced social support. For the nurse-assessed potential predictors (reduced grip strength, poor core stability, inability to own initiative, depressive symptoms and social support), interrater agreement between two independent clinicians was assessed for 13 patients outside the study, and Cohen’s kappa coefficient (κ) was calculated. The assessment of reduced grip strength, core instability, reduced social support and depressive symptoms showed an interrater agreement above 0.9 while inability to take initiative had a κ = 0.69.

#### Assessment of physical disability

As no objective measurement of physical function prior to ICU admission was possible to obtain, patients or their next-of-kin was asked, at ICU discharge, to describe the patient’s physical function based on the Katz ADL Index [14] two weeks prior to hospitalization. The Katz ADL Index measures the patient’s ability to independently manage six basic activities in daily life: hygiene, dressing/undressing, toileting, mobility, continence and food intake. Each activity was evaluated with regard to the patient’s ability to perform the activity independently or not.

To determine the presence of physical disability two months after ICU discharge, surviving patients were sent the ADL staircase questionnaire [15]. The ADL staircase consists of 10 items and is an extended version of the Katz ADL Index. Besides the Katz index’ six items evaluating personal ADL, the ADL staircase contains four items regarded as instrumental ADL; cooking, shopping, transportation and cleaning. Each activity was evaluated with regard to the patient’s ability to perform the activity independently or not.

We defined new-onset physical disability as if a patient (a) had greater dependency in ADLs (required assistance in basic activities) compared to his/her reported functional status prior to ICU admission or (b) had been working prior to ICU admission and was currently on sick leave for physical reasons.

For patients with impaired instrumental ADL as assessed with the ADL staircase two months after ICU discharge and without reported baseline ADL impairment in Katz ADL prior to ICU admission, additional medical chart review and a phone call to the patient was made to confirm that the impairment was new-onset.

### Statistical analysis

Continuous data were presented as means and standard deviations when normally distributed, otherwise medians and interquartile ranges were used. Categorical data were presented with percentages. Student’s *t* test (normal distribution) or Mann Whitney *U* test (in the cases of skewed distribution) were used comparing continuous data between patient characteristics in Table 1 and between responders and nonresponders, while Fisher’s exact test was used to compare categorical data. In the predictive model, only age and SAPS 3 were included as numeric predictors. Continuous variables with skewed distribution (ICU length of stay, duration of sedative, invasive ventilator treatment and fractures) were dichotomized. Univariate associations between potential predictors and adverse outcome were examined to evaluate the potential predictors for new-onset physical disability. The predictors were tested as independent variables in a logistic regression model one at a time. Variables with a *P* value >0.10 were excluded from further analysis. The remaining variables were included in a multivariable logistic regression model. The accuracy of the predictive model was measured as the area under receiver operating characteristics (AUC) curve. The predictors were removed one at the time, and the AUC was recalculated each time [16]. An internal cross-validation in 1,000 bootstrap samples was performed to further evaluate the predictive accuracy of the screening instrument. Calibration of the model was assessed and graphically displayed by plotting observed risk of physical disability against predicted risk of physical disability across 20% risk strata. The analyses were performed using Stata version 12 (StataCorp, College Station, TX, USA) and SPSS version 20.0 (IBM SPSS, Armonk, NY, USA).

## Results

Three hundred eighty-nine patients were discharged from the general ICU during the study period. Thirty-five percent of these patients were excluded, mainly due to language difficulties or because they were discharged to other ICUs. Of 232 eligible patients 148 (64%) completed the questionnaires (Figure 1). Questionnaires responders were older (mean age of 55 versus 47, *P* <0.05) and had more pre-existing diseases (median 1 versus 0, *P* <0.05) compared to nonresponders. One patient was excluded because of a traumatic leg amputation, which was considered an extreme case preventing the patient to return to the pre-ICU status within two months. Among responders, 95% reported no ADL impairment prior to ICU admission. Sixty-nine patients (47%) reported new-onset physical disability, that is having worsened ADL compared to before ICU admission, or being on sick leave due to new-onset physical disability. Patient and treatment characteristics for those with and without new-onset physical disability two months after ICU discharge are provided in Table 1.

### The predictive model

**Figure 2:**
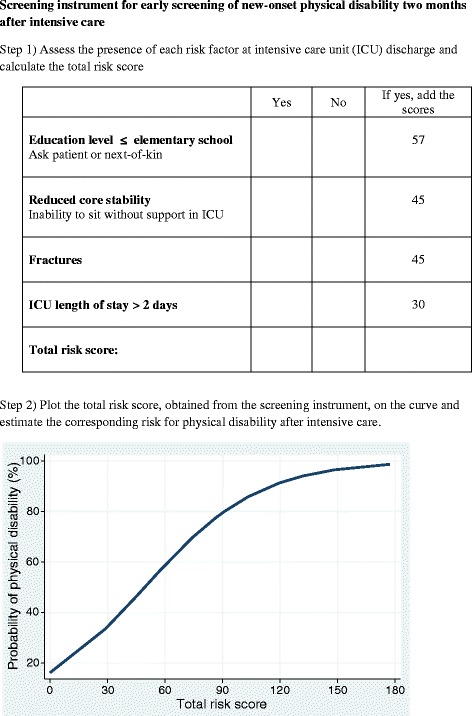
**The predictive screening instrument.**

**Figure 3:**
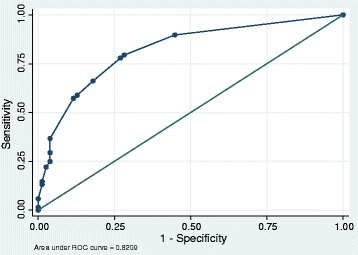
**Area under the receiver operating curve of the predictive model.**

**Figure 4:**
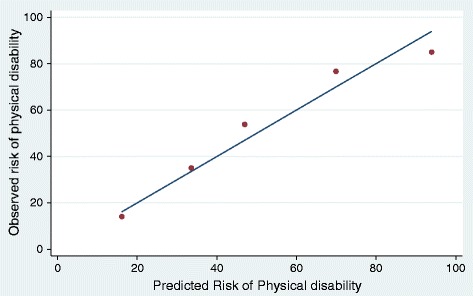
**Calibration curve comparing observed and predicted risk of physical disability across 20% strata.**

A description of the potential predictors and their univariate associations with new-onset physical disability are shown in Table 1. Ten variables were considered sufficiently associated with adverse outcome (*P* <0.1) in the univariate analysis to merit inclusion in the multivariable logistic regression modeling of the prediction instrument (Table 2). Age, SAPS 3, comorbidity, midazolam use, delirium and ability to own initiative were in turn removed from the model as these factors increased the area under the curve by less than one percentage point and thus not considered essential from a clinical viewpoint. The predictors are presented with odds ratios (ORs) and 95% confidence intervals (CI) in Table 2. The final model consisted of four predictors: low educational level, impaired core stability, fractures and ICU stay >2 days. ICU length was dichotomized with a cutoff of two days as this cutoff demonstrated a distinct divergence in the predictive value. Each variable’s contribution in the predictive screening instrument was based on its regression coefficient. To simplify the use of the clinical predictive screening instrument, the coefficients were multiplied by 30 and named ‘risk scores’ (Table 2). The developed predictive screening instrument is shown in Figure 2. The performance of the model presented as the AUC was 0.82 (Figure 3) and the 1,000 bootstrap cross-validated AUC was 0.80 (95% CI 0.69 to 0.90). Figure 4 displays the relationship between observed and predicted risk of physical disability across five risk strata. The predictive accuracy for ICU length of stay alone as a predictor for physical disability (AUC) was 0.70 (95% CI 0.61 to 0.78).

**Table 2:** **Odds ratio and 95% confidence intervals for variables included in the multivariable logistic regression modeling**

**Predictors**	**Odds ratio (95% CI)**	**Regression coefficient**	**Risk score**
Age	1.01 (0.98-1.04)		
Low educational level*	6.79 (2.28-20.22)	1.9	57
Somatic comorbidity	1.67 (0.67-4.16)		
SAPS 3	1.01 (0.98-1.04)		
ICU length of stay >2 days*	2.60 (1.22-5.80)	1.0	30
Midazolam sedation >24 hours	0.73 (0.21-2.62)		
Fractures*	4.48 (1.07-18.75)	1.5	45
Delirium	1.06 (0.38-2.97)		
Impaired core stability*	4.61 (2.02-10.49)	1.5	45
Inability to take initiative	0.89 (0.31-2.54)		

*Regression coefficients and risk scores are presented for predictors included in the final model. ICU, intensive care unit; SAPS 3, Simplified Acute Physiology Score 3.

## Discussion

In this prospective study, we assessed the relative contribution of previously described risk factors in relation to new-onset physical disability in a mixed ICU population and used the information to develop a method for early screening of ICU survivors. To our knowledge, this is the first description of an instrument that enables clinicians to estimate the risk for subsequent new-onset physical problems already at ICU discharge.

Our data demonstrate that predisposing vulnerability as well as ICU-related factors contributes to new-onset physical disability after critical illness. Low educational level was the strongest predictor of new-onset physical disability two months after ICU discharge (OR = 6.8). In previous studies, low educational level has been associated with a higher mortality and morbidity rate [17,18] and poor functional outcome [19]. The difference in coping strategies between educational levels has been suggested to influence outcome after rehabilitation. Patients with low education are suggested to employ more avoidant coping, while highly educated patients tend to use more problem-oriented coping strategies [20]. Problem-solving and goal-setting strategies are characterized by proactive behavior and improved outcome in rehabilitation [21]. Besides coping, active communication appears to be important in rehabilitation after hospital discharge and has been associated with patients’ socioeconomic status [22]. In hospital settings, patients with higher education were found to communicate more actively, expressing their concerns and preferences regarding health status and rehabilitation and received more information than patients with low education [22].

Fractures and impaired core stability are well-described risk factors for long-term physical disability [23,24]. Impaired core stability is likely a consequence of critical illness and the immobilization associated with traditional intensive care. This finding is in line with a recent study of mobilization during daily sedative interruption, in which return to independent functional at hospital discharge was significantly improved by early mobilization during sedation stops [25].

In our literature review prior to the study, ICU length of stay did not show consistent linearity with long-term outcomes. For this reason, and also for greater generalizability of the instrument we chose not to restrict the study to patients with longer ICU length of stay. Consequently, the mean ICU length of stay in the patient cohort was relatively short. In the regression model, ICU length of stay as a risk factor had an odds ratio of 2.6 but was not the most important predictor for physical disability (Table 2).

Despite in-ICU mobilization with physiotherapists available six days per week in our ICU, an intervention demonstrated to improve ADL after ICU stay [25], 47% of the responding patients suffered from physical disability two months after ICU discharge. Considering the absence of structured rehabilitation after hospital discharge in many ICU survivors [26] we find it likely that some patients’ new-onset disability may have been prevented with efficient rehabilitation between ICU discharge and two months post-ICU. The fact that educational level was the strongest predictor indicates that there may be room for improving the trajectory of physical recovery with better patient education or physiotherapy sessions. However, a study targeting high-risk patients is needed to confirm this hypothesis.

The timing of our outcome measure, reduced ADL compared to ADL before ICU admission, can be discussed. In some patients with severe injury or illness, full ADL recovery two months after ICU discharge after critical illness may be unlikely despite optimal rehabilitation. Considering that low educational level was the strongest predictor of ADL reduction two months post-ICU however, we have reason to believe that recovery to pre-ICU ADL at two months can potentially be modified in a substantial proportion of patients.

### Practical use of the predictive screening instrument

To calculate the patient’s risk for physical disability, the presence of predictors in the patient at the time for discharge to the ward is assessed (Figure 2). Each predictor renders a risk score as shown in Table 2. The individual risk scores are added together to a total risk score. For example, a patient with a fracture and an ICU length of stay of four days has a total risk score of 45 + 30 = 75. The total risk score can then be plotted on the risk probability curve, which in this example corresponds to a probability for physical disability of approximately 75%. Triage based on risk estimation may enable resource allocation for ICU follow-up, by for example concentrating intensified post-ICU physiotherapy interventions to patients with high risk for physical problems.

The screening instrument is to some extent similar to the described ‘short clinical assessment’ and ‘functional assessment’ in the NICE guidelines. However, a major difference is that the screening instrument renders a risk as a percentage that may be useful for clinicians in decision-making for follow-up or not.

### Limitations

The main limitation of our study is that the screening instrument was developed through evaluation of ICU patients in a single tertiary care hospital with a limited number of participants and the instrument is not externally validated. Thus, generalization of our results to other populations must be made with caution. With this stated, our cohort consisted of mixed medical and surgical patients, which we believe makes our findings applicable to a large group of ICU survivors. In lack of validated tools appropriate for use at ICU discharge, the potential predictors’ ability to initiative, depressive symptoms and social support were assessed with proxy measures. While interrater agreement was good in our setting, a validated method for assessing these predictors would have been preferred and might have improved the accuracy of the measurement.

Objective baseline measurements are difficult or not possible in patients with emergency admissions to the ICU. While retrospective reporting of functional status has been found to correlate with objective findings [27], we acknowledge the risk of recall bias as patients were asked to estimate their pre-ICU ADL status after ICU admission. In our study, 95% of patients rated no functional (ADL) impairment prior to ICU admission; information of a distinct character we believe is likely to be easier to recall than to rate intermediate functional levels despite retrospective reporting. Different ADL scales were used to assess functional status pre- (Katz ADL index) and post-ICU (ADL staircase). Optimally, the same instrument would have been used for both pre- and post-ICU status. In planning the study, the ADL staircase was considered to be too cumbersome to administer to patients at ICU discharge.

Some previous ICU follow-up studies have shown an association between physical disability and cognitive dysfunction [19]. We assessed cognitive function by monitoring delirium in the ICU, which was more common in patients with adverse outcome but did not contribute to the prediction of adverse outcome. We did not assess cognitive function after the ICU stay. Cognitive dysfunction in the ward, after ICU discharge may have played a role in the trajectory of physical recovery and rehabilitation but was not monitored, as the intention was to develop a screening instrument for use *at* ICU discharge.

## Conclusions

A screening instrument for early, in-ICU prediction of new-onset physical disability two months after ICU discharge has been developed. The instrument includes four risk factors for assessment and has fair predictive power (cross-validated AUC 0.80). The instrument may be useful in identifying ICU survivors in need of follow-up. Moreover it may improve the power to detect potential benefits of early interventional follow-up in ICU survivors but needs external validation before use in other populations.

## Key messages

- Low educational level, impaired core stability at ICU discharge, the presence of fractures and prolonged ICU stay appear to influence the trajectory of recovery after critical illness.
- A simple screening instrument based on these predictors enables clinicians to estimate the risk for subsequent new-onset physical problems already at ICU discharge.

## Additional file

Additional file 1:
**This file describes in detail the identified risk factors for physical morbidity in the literature review [** 3 **,** 19 **,** 28 **-** 37 **].**

## Abbreviations

ADL: activity of daily living
AUC: area under the receiver operating characteristics curve
BMI: body mass index
CCI: Charlson Comorbidity Index
CI: confidence interval
ICU: intensive care unit
OR: odds ratio
SAPS 3: Simplified Acute Physiology Score 3
