# Supplementary material for: Early prediction of new-onset physical disability after intensive care unit stay: a preliminary instrument
Source: Crit Care. 2014 Jul 31;18(4):455. doi: 10.1186/s13054-014-0455-7 (PMC4243809; doi:10.1186/s13054-014-0455-7)
Supplement: Additional file 1: — This file describes in detail the identified risk factors for physical morbidity in the literature review [ 3 , 19 , 28 - 37 ]. [file 13054_2014_455_MOESM1_ESM.docx]

**ADDITIONAL FILE 1.**

**Table. Identified risk factors for physical disability after ICU or hospital stay in the literature review and potential risk factors included in the prediction study**

| **Study** | **Identified risk factors** | **Potential predictors in the study** |
| --- | --- | --- |
| Miller et al.[28] | Increasing age High body mass Low income Worse self-rated ADL and health Cognitive impairment Fractures Somatic co-morbidity Depression Severity of illness Length of stay in hospital Less social activity | Included Included Not assessed – educational level assessed Included Assessed as ICU delirium Included Included Included Included as SAPS 3 Difficult to assess at ICU discharge Difficult to assess at ICU discharge |
| Schweickert et al.[29] | Systemic inflammation Corticosteroid treatment High blood glucose level Immobility Duration of mechanical ventilation | Included Considered not practicable to assess Considered not practicable to assess Included Included |
| Mock et al.[30] | Increasing age Socioeconomic status Preinjury health Social support | Included Included Included Included |
| De Letter et al.[31] | Severity of illness SIRS | Included as SAPS 3 Included |
| Nanas et al.[32] | Aminoglycosides Severity of illness Hyperglycemia Gram (−) bacteremia | Considered not practicable to assess Included as SAPS 3 Considered not practicable to assess Considered not practicable to assess |
| De Jonghe et al.[33] | Multiorgan failure Immobility Hyperglycemia Medication | Included Included Considered not practicable to assess Considered not practicable to assess |
| Ely et al.[13] | Delirium (risk factor for prolonged hospital stay) | Included |
| Holbrook et al.[35] | Postinjury depression Post-traumatic stress disorder Severity of injury ICU length of stay | Assessed as in-ICU depressive symptoms Not possible to assess at ICU discharge Included Included |
| Chaudhry et al.[19] | Low educational level Cognitive impairment Lack of social support High co-morbidity Poor self-related health | Included Difficult to assess at ICU discharge Included Included Not possible to assess at ICU discharge |
| Latronico et al.[36] | Sepsis/SIRS Multiorgan failure Prolonged mechanical ventilation Vasopressor and catecholamines Severity of illness Prolonged ICU stay Hyperglycemia Female gender Renal failure Hyperosmolality Parenteral nutrition Low serum albumin Neurological failure Immobility | Included Included Included Considered not practicable to assess Included as SAPS 3 Included Considered not practicable to assess Included Considered not practicable to assess Considered not practicable to assess Considered not practicable to assess Considered not practicable to assess Considered not practicable to assess Included |
| Stevens et al.[37] | Female gender Severity of illness Sepsis/SIRS Renal replacement therapy High blood glucose level Total parenteral nutrition Pharmacological exposure | Included Included as SAPS 3 Included Considered not practicable to assess Considered not practicable to assess Considered not practicable to assess Considered not practicable to assess |
| The NICE guidelines [39] | Prolonged ICU stay Unable mobilize independently Profound physical or neurological injury Cognitive impairment Increased oxygen demand Reduced ADL function pre-ICU | Included Included Proxy SAPS 3 and fractures Assessed as ICU delirium Included Included |
